# Breakthroughs in immune checkpoint therapy: overcoming NSCLC immune checkpoint therapy resistance with novel techniques

**DOI:** 10.3389/fimmu.2025.1630940

**Published:** 2025-09-02

**Authors:** Li-Ping Kang, Hua-Jing Huang, Cong Xu, Hui-Hui Chen, Dong-Hui Huang, Ze-Bo Jiang

**Affiliations:** ^1^ Zhuhai Hospital of Integrated Traditional Chinese & Western Medicine, Zhuhai, Guangdong, China; ^2^ Northeastern University, Boston, MA, United States; ^3^ Department of Oncology, The Affiliated Cancer Hospital of Nanjing Medical University & Jiangsu Cancer Hospital & Jiangsu Institute of Cancer Research, Nanjing, China

**Keywords:** immune checkpoint inhibitors, NSCLC, resistance mechanisms, combination immunotherapy, epigenetic modifiers, personalized neoantigen vaccines, microbiome signatures, T-cell inflamed gene signature

## Abstract

Immune checkpoint therapy has emerged as a revolutionary approach in the field of non-small cell lung cancer (NSCLC), offering new hope to patients with various malignancies. Despite its success, a significant proportion of patients exhibit primary or acquired resistance, limiting the efficacy of these treatments. This review provides a comprehensive analysis of recent breakthroughs in immune checkpoint therapy, focusing on the underlying biology of immune checkpoints, current checkpoint inhibitors, and the mechanisms of resistance that challenge treatment effectiveness. In particular, we will explore novel strategies designed to overcome these resistance mechanisms, including combination therapies that enhance anti-tumor immune responses, the use of personalized neoantigen vaccines, and microbiome-modulating therapies. Additionally, we will examine the role of emerging biomarkers, such as TCR clonality and T-cell inflamed gene signatures, in predicting patient responses. By synthesizing these insights, this review aims to highlight innovative approaches that could significantly improve therapeutic outcomes for patients with NSCLC and other malignancies, ultimately advancing the field of cancer immunotherapy.

## 1 Introduction

Lung cancer is the leading cause of cancer-related mortality worldwide, with non-small cell lung cancer (NSCLC) constituting the most prevalent histological subtype, accounting for approximately 80-85% of all cases (1, 2). Despite a range of treatment options, including surgery, radiotherapy, chemotherapy, and immunotherapy, survival rates for advanced-stage NSCLC patients remain disappointingly low (3). Standard chemotherapy regimens often lead to severe side effects and the development of drug resistance, which has prompted a shift toward targeted therapies. However, tumors frequently acquire resistance to these targeted treatments over time, complicating therapeutic efficacy (4, 5).

In recent years, innovative cancer therapies such as adoptive cell therapies, immune checkpoint inhibitors (ICIs), and cancer vaccines have demonstrated promising efficacy in treating NSCLC (6–8). For example, certain therapies can enhance anti-tumor immune responses by inhibiting or downregulating programed cell death-1 (PD-1) on effector CD8^+^ T cells or regulatory T cells (Tregs). Nonetheless, only a fraction of NSCLC patients experiences significant benefits from these treatments, while many others develop resistance, highlighting the need for improved strategies.

The advent of ICIs, including anti-PD-1, anti-PD-L1, and anti-CTLA-4 antibodies, represents a landmark shift in cancer therapy. These agents harness the immune system’s inherent capability to recognize and eradicate tumors (9). Despite these remarkable advancements, a substantial proportion of patients either demonstrate no response to ICIs or experience resistance after an initial positive response (10). Understanding the underlying mechanisms of resistance to these therapies is essential for improving clinical outcomes and enhancing the efficacy of treatments.

To address these challenges, emerging hot topics in the field warrant attention, particularly the role of artificial intelligence (AI) and machine learning in predicting patient responses to ICIs (11). These technologies analyze complex datasets and identify biomarkers that can more accurately predict which patients are likely to benefit from immunotherapy, thus enabling personalized treatment approaches. Tumor mutational burden (TMB) and microsatellite instability (MSI) have also garnered significant interest as predictive biomarkers, serving as vital tools in stratifying patients who may respond favorably to ICIs (12). Additionally, the exploration of microbiome-modulating therapies offers exciting new possibilities for enhancing ICIs efficacy (13, 14). Recent studies have shown that gut microbiomes can influence immune responses, affecting how patients respond to immunotherapy and potentially guiding treatment decisions (15). By integrating insights from AI, novel biomarker identification, and microbiome research, the field can develop more effective strategies to overcome resistance and optimize the benefits of immunotherapy for a broader range of patients. Identifying novel strategies to overcome these barriers is critical to advancing the frontier of NSCLC treatment and ensuring that more patients can benefit from the potential of immunotherapy. In this review, we aim to provide an in-depth analysis of recent breakthroughs in immune checkpoint therapy, focusing on novel techniques to overcome resistance mechanisms. We will discuss the underlying biology of immune checkpoints, the current landscape of checkpoint inhibitors, the mechanisms of resistance, and innovative strategies being explored to enhance therapeutic outcomes.

## 2 Mechanisms of immune checkpoint therapy

### 2.1 The immune system and tumor immunology

The immune system plays a pivotal role in the detection and elimination of malignant cells, a process known as tumor surveillance. This complex network involves various immune cell types, including T cells, B cells, natural killer (NK) cells, dendritic cells, and macrophages (16). These components work in concert to identify and destroy cells that exhibit aberrant characteristics, such as mutations or altered antigen expression (17). Through ongoing surveillance, the immune system can often target nascent tumors before they develop into clinically detectable disease.

**Figure 1:**
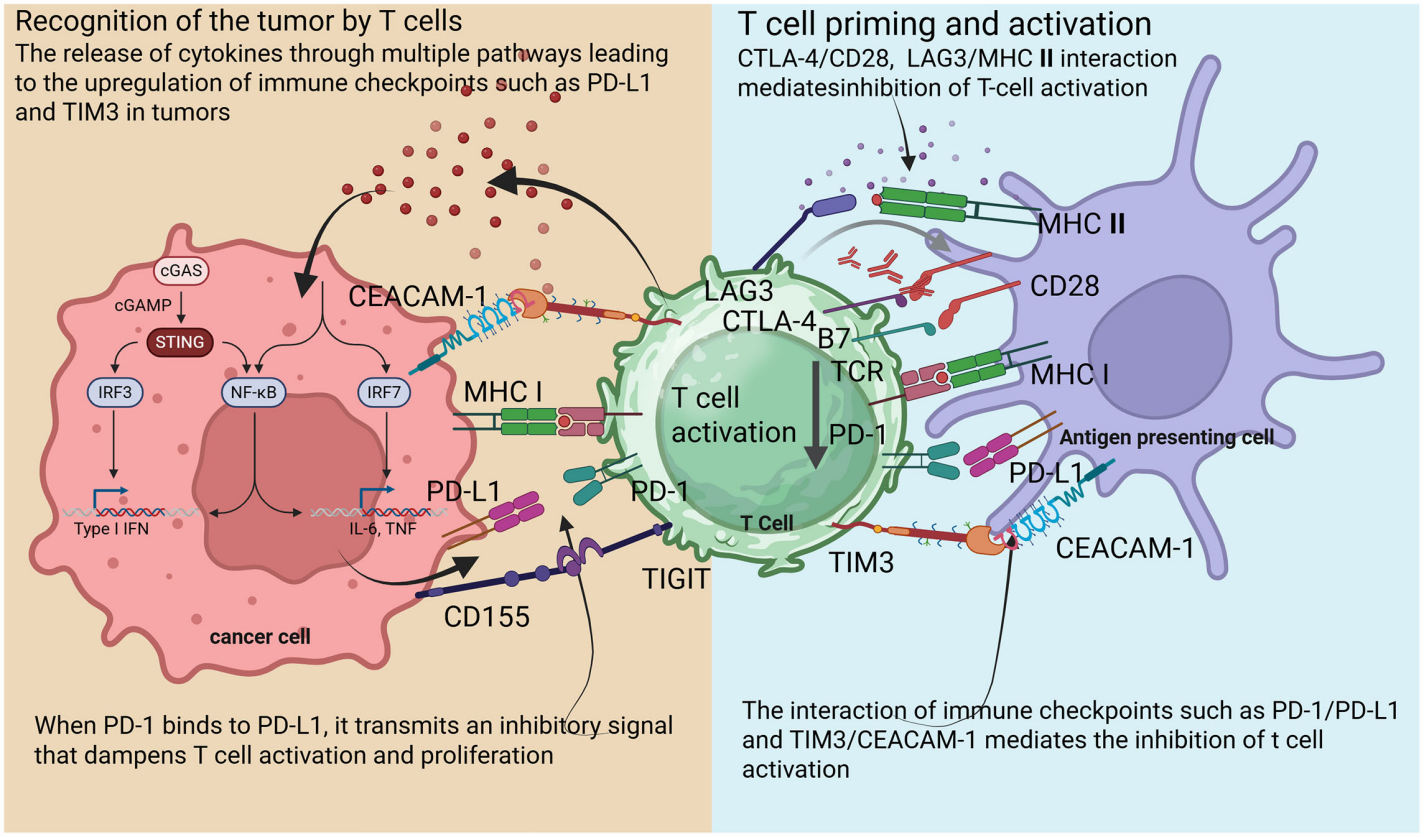
Schematic diagram illustrating mechanisms of T cell activation and suppression in the tumor microenvironment.

Central to the immune response are immune checkpoints, which are regulatory molecules that modulate T cell activity (18). These checkpoints maintain homeostasis within the immune system, preventing excessive responses that could lead to autoimmunity (**Figure 1**) (19). Key checkpoints include PD-1, programmed cell death ligand-1 (PD-L1), and cytotoxic T lymphocyte-associated protein 4 (CTLA-4) (20). The PD-1 pathway, in particular, represents a critical checkpoint in T cell regulation. PD-1 is expressed on the surface of activated T cells, Treg cells and macrophages, while its ligands, PD-L1 and PD-L2, are often overexpressed on tumor cells and in the tumor microenvironment (TME) (21). When PD-1/PD-2 binds to PD-L1 on tumor cells, it sends an inhibitory signal that dampens T cell activation and proliferation, allowing tumors to evade immune detection (22).

The checkpoint molecule CTLA-4 competes with the co-stimulatory receptor CD28 for binding to B7–1 and B7–2 on antigen-presenting cells (APCs) (23). This negative regulation occurs early in T cell activation and serves to modulate immune responses, thereby preventing autoimmunity (24). ICIs enhance T cell activation by blocking these pathways (25). TME plays a critical role in shaping the immune response to tumors. This complex ecosystem encompasses not only tumor cells but also various immune cells, stromal components, and the extracellular matrix (26). TME can influence both tumor susceptibility to immune attack and the capacity of immune cells to mount effective responses (27).

Within the TME, several immunosuppressive mechanisms are at play, including recruitment of Tregs, secretion of immunosuppressive cytokines (e.g., IL-10, TGF-β), and the presence of myeloid-derived suppressor cells (MDSCs) (28). Furthermore, metabolic changes within the TME can further inhibit T cell function. Tumors often exhibit altered metabolic pathways that lead to the accumulation of adenosine, a metabolite known to suppress T cell activation (29, 30). Targeting these immunosuppressive elements within the TME represents a promising strategy to enhance the efficacy of checkpoint blockade therapies (31).

The immune system utilizes checkpoints to regulate responses, preventing autoimmunity while enabling the detection of malignant cells (32). ICIs disrupt these regulatory signals, unleashing a potent anti-tumor immune response (9). T cell exhaustion, characterized by a progressive loss of effector functions, often results from chronic antigen exposure, such as in tumors (33). Exhausted T cells exhibit elevated levels of inhibitory receptors, including PD-1, CTLA-4, and TIM3, along with reduced proliferation and cytokine production (34).

Emerging evidence indicates that ICIs can restore the functionality of exhausted T cells, enabling them to regain their immune activity (10, 35, 36). However, the clinical efficacy of these agents can be highly heterogeneous. While some patients experience improved survival outcomes, others may not respond or exhibit subsequent relapses following initial benefits (37–39). This variability underscores the need for a deeper understanding of the mechanisms driving both responses and resistance to ICIs.

Current research focuses on identifying biomarkers capable of predicting responses, regulating the TME, and exploring combination strategies (40–42). Combining ICIs with other modalities—such as chemotherapy, targeted therapy, or novel agents that modulate the TME (43–45)—has shown promise in preclinical and clinical studies (46–49). Integrating AI and machine learning to analyze large datasets offers a powerful approach for predicting patient responses based on genetic and epigenetic profiles, including biomarkers like TMB and MSI (50). Furthermore, novel therapies that modulate microbiomes are being investigated for their potential to enhance ICI efficacy by influencing the immune landscape (51). By addressing the multifaceted mechanisms of immune evasion and optimizing treatment regimens, it may be possible to improve outcomes for a broader range of patients (52). In conclusion, immune checkpoint therapy has revolutionized the approach to cancer treatment by leveraging the body’s immune defense mechanisms. Understanding the intricacies of the immune system, checkpoint regulation, and tumor biology are fundamental for further advancements in this field. As research continues to unravel the complexities of immune evasion and response, there is hope for optimizing ICI therapies and improving the prognosis for patients with cancer.

### 2.2 Key immune checkpoints

The advent of immunotherapy has profoundly transformed cancer treatment, especially with the development of ICIs that capitalize on the immune system’s capacity to recognize and eliminate malignant cells by targeting immune checkpoint pathways exploit by tumor to evade immune detection and destruction (53). A nuanced understanding of key immune checkpoints, particularly the PD-1/PD-L1 pathway and CTLA-4, is essential to appreciate how ICIs enhance anti-tumor immunity. In this review, we will explore the mechanisms of these critical checkpoints, their role in tumor biology, and the significant clinical impacts of ICIs in cancer therapy.

#### 2.2.1 PD-1/PD-L1 pathway

The PD-1/PD-L1 pathway is critical for immune evasion. Tumors often express PD-L1, which binds to PD-1 on T cells, leading to T cell inactivation (54). ICIs enhance T cell activity against tumors by targeting this pathway, which plays a key role in regulating immune responses and tumor evasion (55). PD-1 is an inhibitory receptor found on activated T cells, while PD-L1 is a ligand that can be upregulated on tumor cells within TME (56). When PD-1 binds to PD-L1, it transmits an inhibitory signal that dampens T cell activation and proliferation (57). While this interaction is essential for maintaining immune tolerance and preventing autoimmunity, tumors harness it to escape immune surveillance. Tumor cells may express PD-L1 constitutively or in response to inflammatory cytokines, such as IFN-γ and TNF-α, within the TME (58).

**Figure 2:**
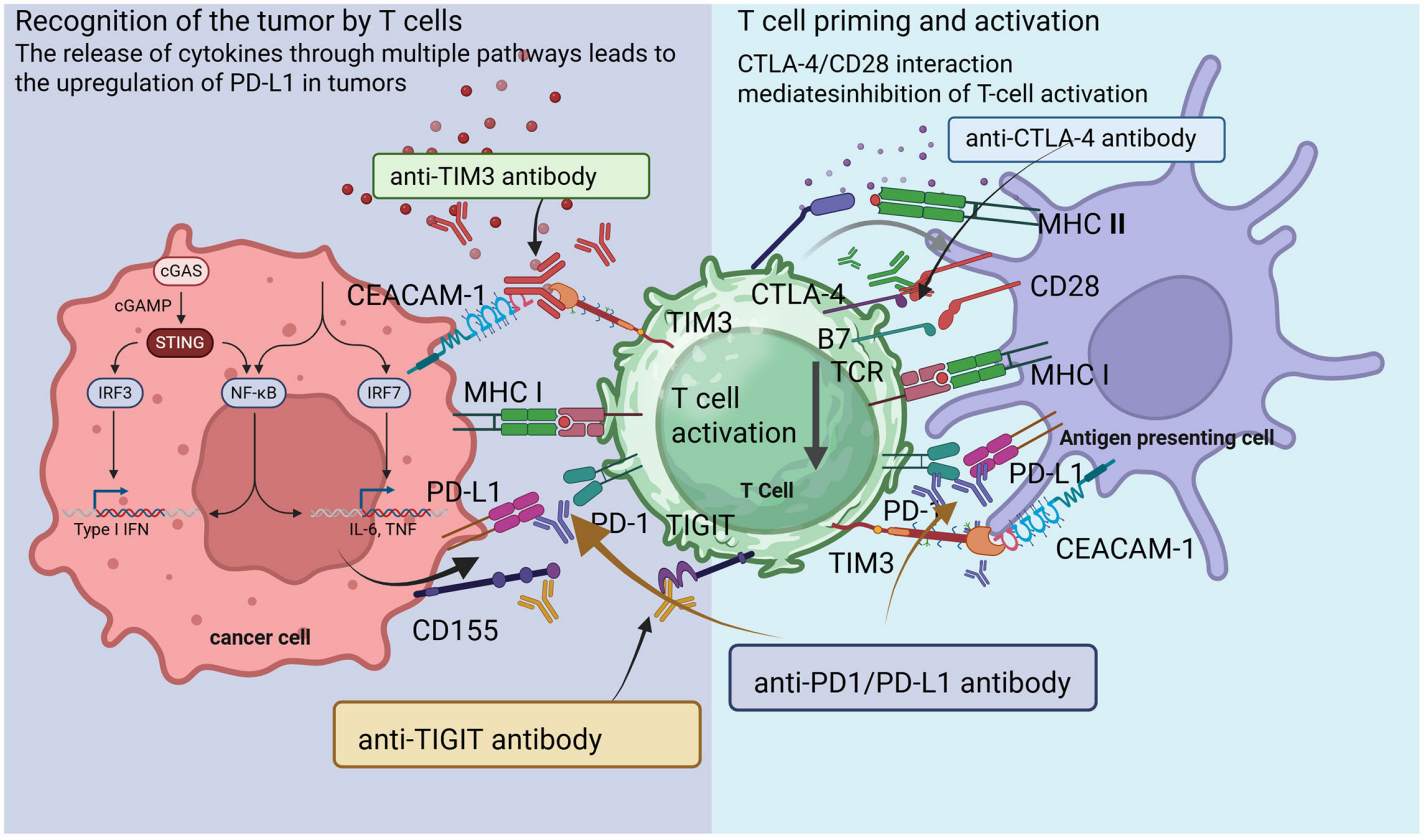
Mechanisms of immune checkpoint inhibitors.

While this interaction is essential for maintaining immune tolerance and preventing autoimmunity, tumors harness it to escape immune surveillance. Tumor cells may express PD-L1 constitutively or in response to inflammatory cytokines, such as IFN-γ and TNF-α, within the TME (59). The binding of PD-L1 to PD-1 on effector T cells reduces the production of key cytokines, including IFN-γ and interleukin-2 (IL-2), which are crucial for T cell activation (60). By silencing T cells, this immune checkpoint permits tumors to proliferate unchecked and evade immune-mediated destruction (61). Notable examples include pembrolizumab and nivolumab (PD-1 inhibitors) and atezolizumab and durvalumab (PD-L1 inhibitors) (**Figure 2**) (62–65).

Recent clinical trials have demonstrated that PD-1/PD-L1 blockades can lead to durable responses in diverse malignancies, including melanoma, NSCLC, and renal cell carcinoma (66). Key findings from these studies show significant improvements in overall survival and progression-free survival rates compared to traditional treatments (67). For instance, nivolumab has been associated with long-term survival rates in patients with advanced NSCLC (68). However, not all patients benefit from these therapies. Resistance mechanisms can be broadly categorized into intrinsic tumor factors and extrinsic factors related to the immune microenvironment (69). Tumors may downregulate antigen presentation or upregulate other inhibitory pathways, further complicating T cell activation (70). Additionally, the presence of immunosuppressive cells like Tregs and MDSCs in the TME can inhibit T cell function, leading to resistance (71). Ongoing research aims to identify predictive biomarkers for patient selection and develop combination therapies to overcome resistance. For example, combining PD-1 inhibitors with other modalities, such as CTLA-4 blockade or targeted therapies, has shown promise in enhancing therapeutic efficacy and mitigating resistance (72).

#### 2.2.2 CTLA-4

Cytotoxic T lymphocyte-associated protein 4 (CTLA-4) is another critical immune checkpoint that regulates T cell activation (73). Expressed shortly after T cell activation, CTLA-4 serves as an inhibitory receptor that limits T cell responses (74). It competes with the co-stimulatory receptor CD28 for binding to B7-1 (CD80) and B7-2 (CD86) on APCs (75). CTLA-4 ligation transmits a negative signal that inhibits T cell activation and proliferation. By dampening the immune response, CTLA-4 plays a crucial role in maintaining peripheral tolerance and preventing autoimmune reactions (76–78). However, this regulatory function can be exploited by tumors to downregulate the anti-tumor immune response.

Therapeutic blockade of CTLA-4 with monoclonal antibodies like ipilimumab has been employed successfully in various malignancies (79). Inhibiting CTLA-4 restores the signaling required for T cell proliferation, leading to enhanced T cell activity against tumors (80). Ipilimumab was the first ICI approved by the FDA for metastatic melanoma, paving the way for subsequent checkpoint blockade research (81). Clinical studies have demonstrated significant effects of CTLA-4 blockade, resulting in improved overall and progression-free survival in NSCLC and other cancers (73, 82, 83).

These findings underscored the importance of T cell co-stimulation and the potential to shift the immune response toward a more effective anti-tumor profile. The synergistic effects of combining ipilimumab with PD-1 blockade have also been a major focus, as such combinations can lead to improved response rates and extended survival (84, 85). While CTLA-4 blockade offers therapeutic benefits, it is associated with unique immune-related adverse events (irAEs) (86, 87). Enhanced T cell activation increases the risk of aberrant immune responses that may damage healthy tissues, leading to irAEs such as colitis, dermatitis, and endocrinopathies, often necessitating monitoring and immunosuppressive therapy (88). The variability in patient responses to CTLA-4 inhibition highlights the need for research into biomarkers that predict treatment efficacy and susceptibility to irAEs (89). Future strategies may involve optimizing CTLA-4 blockade, adjusting dosing regimens, or identifying biomarkers indicating which patients are likely to benefit from this intervention.

### 2.3 Clinical impact of ICIs

The FDA has approved numerous ICIs for lung cancer and other malignancies, demonstrating significant improvements in overall survival for select patients (90). ICIs have fundamentally transformed cancer treatment paradigms, leading to several approval milestones for various tumor types, including melanoma, lung cancer, urothelial carcinoma, and head and neck squamous cell carcinoma (91–95). These ICIs have demonstrated consistent clinical benefits, including improved overall survival, prolonged progression-free survival, and enhanced quality of life for many patients, particularly those with advanced disease who previously had limited treatment options (96).

The clinical impact of ICIs extends beyond traditional treatment modalities. In cancers like NSCLC, where outcomes were previously heavily dependent on chemotherapy, the introduction of PD-1/PD-L1 inhibitors has dramatically shifted prognosis (97). Studies have shown that patients treated with these ICIs experience significant survival benefits, often with a lower incidence of severe toxicities compared to conventional chemotherapy (98). These findings establish immune checkpoint therapy not only as a viable option for patients but also as a cornerstone of modern oncological treatment.

However, research and clinical experience indicate that the efficacy of ICIs is limited to 30-40% of patients. Some patients may experience initial benefits, but over time, the therapeutic effect may wane, leading to disease progression. This phenomenon is largely due to the emergence of drug resistance, which limits the broader application of ICIs.

## 3 Resistance mechanisms to immune checkpoint therapy

Despite the promise of ICIs, a considerable number of patients do not respond or develop subsequent resistance following an initial therapeutic benefit. Understanding the mechanisms of both primary and acquired resistance to ICIs is crucial for maximizing their efficacy and improving clinical outcomes (99). The heterogeneity of response rates further underscores the need for a comprehensive understanding of these resistance mechanisms to inform the identification of predictive biomarkers (41).

Resistance mechanisms can be broadly categorized into primary and acquired resistance. Well-studied biomarkers associated with response to ICIs include PD-L1 expression levels, TMB, and MSI (100–104). Patients with tumors exhibiting higher PD-L1 expression or TMB often demonstrate enhanced sensitivity to PD-1/PD-L1 blockade (104–106). However, biomarkers are not universally predictive; some patients with low PD-L1 expression or low TMB still experience clinical responses (107). This inconsistency has fueled research into additional predictive markers, including lymphocyte infiltration, the presence of specific immune subtypes, and genomic profiling.

As our understanding of the immune response to tumors evolves, refining how we stratify patients for ICI therapy is vital to maximizing clinical efficacy. ICIs have transformed cancer treatment, providing significant clinical benefits for a select group of patients across various malignancies (108, 109), the challenge remains to dissect the resistance mechanisms that hinder the efficacy of immune checkpoint therapy. An in-depth exploration of each mechanism will enhance our understanding of tumor biology and the complexity of the immune landscape. By addressing these challenges, future therapeutic strategies can be developed to overcome resistance and optimize the use of ICIs in clinical practice.

### 3.1 Primary resistance

**Figure 3:**
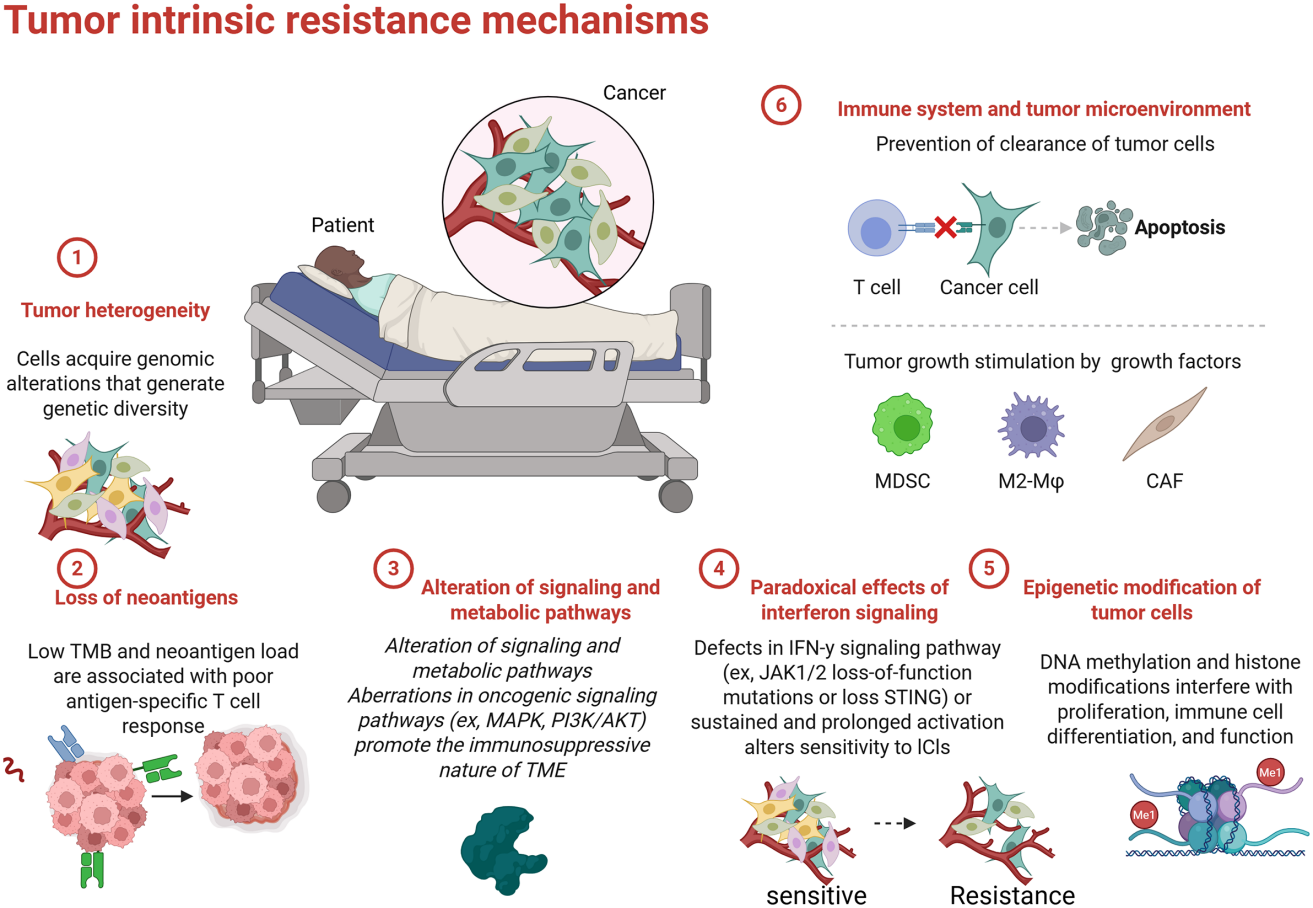
Primary resistance of ICIs.

Primary resistance to ICIs is characterized by a lack of therapeutic response in patients with NSCLC (**Figure 3**) (110). This complex phenomenon is influenced by the tumor’s molecular and immunological features as well as the composition of the TME. Understanding the factors that contribute to primary resistance is essential for developing strategies to enhance the efficacy of ICIs.

The TME plays a critical role in shaping the immune response and determining the effectiveness of ICI therapies (111). Various immunosuppressive cells, such as MDSCs and Tregs, can hinder T cell activation and inhibit anti-tumor immunity (112, 113). MDSCs, a heterogeneous population of myeloid cells that expand in cancer, impede T cell responses by producing immunosuppressive factors, such as arginase, nitric oxide, and reactive oxygen species (ROS) (114). These factors can induce T cell apoptosis, promote T cell anergy, and inhibit the activity of effector T cells. Accumulation of MDSCs in the TME is often associated with poor prognosis and may contribute to primary resistance to ICIs (115).

Tregs are another critical component of the immunosuppressive TME (116). While they maintain peripheral tolerance and prevent overt immune responses. Tregs protect tumors from immune attack by suppressing effector T cell responses characterized by high expression of PD-L1 or CTLA4. Elevated levels of Tregs in the TME signify an unfavorable prognosis and can hinder T cell activation in responses to ICIs (117). The immunosuppressive TME is further shaped by the secretion of cytokines. For instance, the secretion of transforming growth factor-beta (TGF-β) and interleukin-10 (IL-10) can lead to immunosuppressive conditions, thereby reducing T cell function and promoting primary resistance to ICIs (118).

The genetic and molecular characteristics of tumors significantly impact their immunogenicity and response to immune checkpoint therapy. Tumors with low mutation burdens and specific genetic alterations typically exhibit reduced neoantigenicity, impairing immune recognition (119). TMB, defined as the total number of mutations per megabase of DNA within a tumor genome (120), is associated with immunogenicity. High TMB tumors are generally more likely to produce neoantigens that can trigger an immune response (121). Conversely, tumors with low TMB, such as those harboring specific driver mutations or exhibiting chromosomal stability, tend to produce fewer neoantigens, resulting in reduced immune recognition and often leading to poor responses to ICIs (120, 122).

Importantly, recent studies have shown that some patients with low PD-L1 expression and TMB still demonstrate significant responses to ICIs (123). This observation suggests the need for exploring alternative predictive models beyond TMB and PD-L1. The T-cell inflamed gene signature (TIS), characterized by the presence of immune genes indicative of T cell activation, has emerged as a promising alternative marker (124). Higher TIS scores are associated with better responses to therapy, underscoring the importance of a robust intertumoral T cell presence that can potentially override low PD-L1 and TMB levels (125). Tumors may also employ various strategies to evade immune detection, significantly contributing to primary resistance. For instance, downregulation of major histocompatibility complex (MHC) molecules on tumor cells severely limits the presentation of tumor antigens to T cells, impairing T cell-mediated recognition and targeting by the immune system (126). Alongside TME factors and genetic profiles, tumors may utilize alternative mechanisms, such as reduced expression of co-stimulatory signaling molecules and secretion of inhibitory cytokines, to avoid immune detection (127).

Recent research has revealed novel mechanisms of immune escape that further illuminate additional layers of complexity in primary resistance. For instance, studies have shown that tumors utilize the cGAS-STING pathway to subvert immune responses (128). Specifically, adenylate succinate lyase (ADSL) is implicated in the suppression of cGAS-STING signaling, which is crucial for activating innate immune responses (129). ADSL interferes with the synthesis of cyclic GMP-AMP (cGAMP), a critical downstream mediator of the cGAS-STING pathway (130). By downregulating this pathway, tumors can evade detection by the innate immune system, facilitating immune evasion and primary resistance. Targeting ADSL to restore STING pathway activation represents a promising therapeutic strategy that could enhance immune surveillance and improve patient responses to ICIs (129).

Additionally, the role of N-acetyltransferase 8-like (NAT8L) and its metabolite N-acetylaspartic acid (NAA) has been identified in regulating protein acetylation, influencing tumor cell metabolism, and facilitating immune evasion (131). Elevated levels of NAT8L in the TME have been linked to reduced immune activity, thus contributing to therapeutic resistance (132). By targeting NAT8L with specific inhibitors in combination with ICIs, it may be possible to restore acetylation patterns conducive to improved anti-tumor immunity and overcome resistance (133, 134).

Furthermore, tumors may also lose the expression of specific targetable antigens normally recognized by the immune system (133). This antigenic variability can result from selective pressure exerted by immune responses, allowing the emergence of subclonal populations that do not express prominent tumor antigens, resulting in a lack of recognition by T cells (135). Additionally, tumor cells can downregulate or lose MHC class I expression, impeding T cell recognition and activation (136), thereby granting a survival advantage against immune-mediated destruction (137). The upregulation of soluble PD-L1 in the TME can also inhibit CD28-mediated co-stimulation, further complicating T cell activation and contributing to primary resistance (138, 139). In summary, understanding the mechanisms behind ADSL-mediated cGAS-STING suppression and the role of NAT8L/NAA in immune escape offers new avenues for therapeutic intervention. Targeting these pathways may enhance the efficacy of ICIs and improve outcomes for patients with primary resistance to treatment.

### 3.2 Acquired resistance

**Figure 4:**
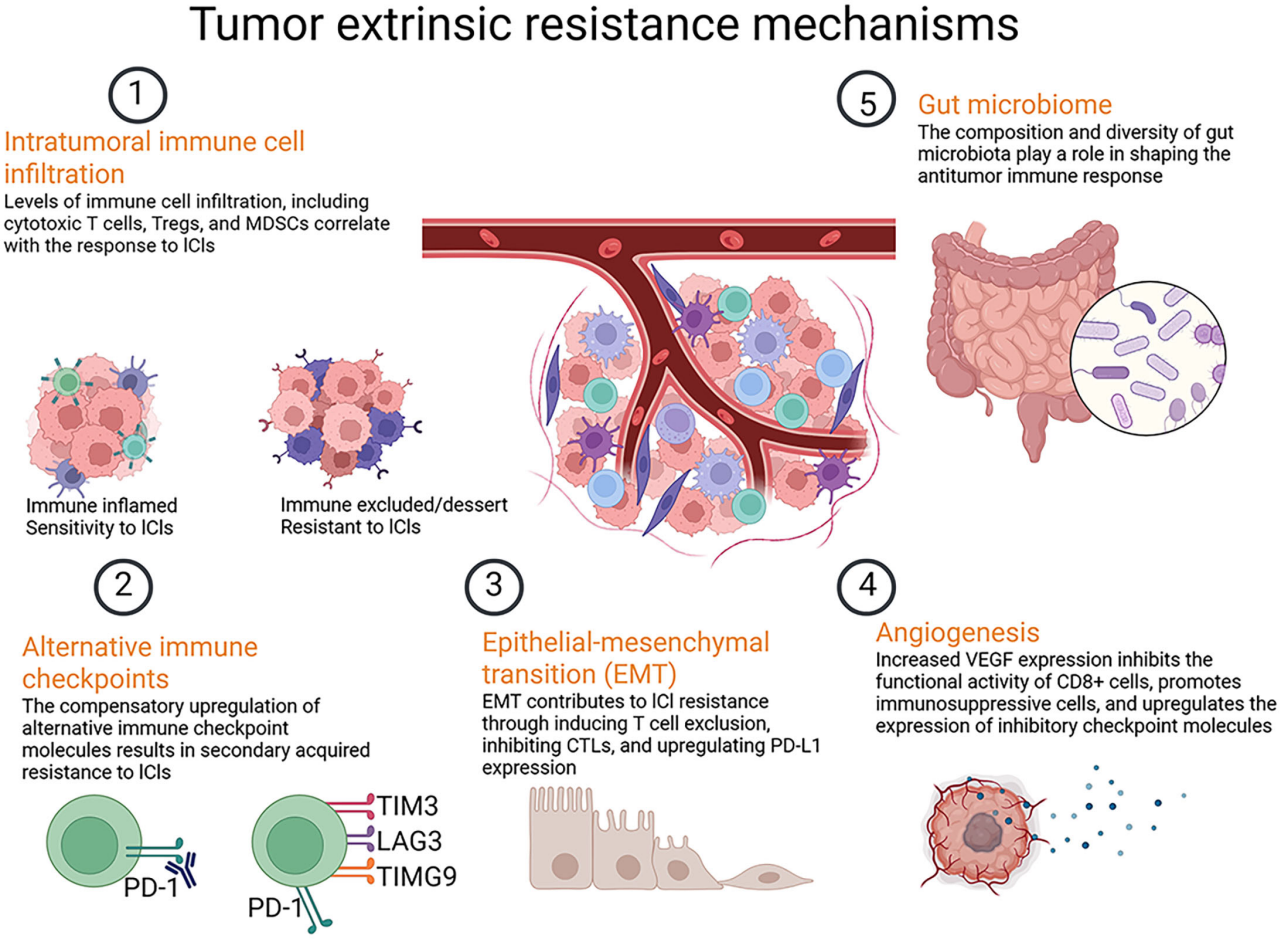
Tumor extrinsic resistance mechanisms to ICIs.

Acquired resistance develops in patients who initially respond to ICI therapy but subsequently exhibit a decline in therapeutic efficacy. This phenomenon underscores the dynamic interplay between the immune system and tumor evolution, revealing how tumors adapt to immune pressures over time (140). One prominent mechanisms of acquired resistance involve alteration in the tumor’s antigenic profile (141, 142). Similar to primary resistance, acquired resistance can occur when tumors lose expression of antigens targeted by ICIs (**Figure 4**) (143). Tumors that initially express high levels of antigens targeted by ICIs may evolve to downregulate or eliminate these antigens in response to immune pressure, preventing T cell re-recognition (144) (**Figure 4**).

Analysis of tumor biopsies has shown that acquired resistance may stem from defects in interferon-gamma (IFN-γ) signaling pathways. For example, acquisition of JAK mutations represents an early step in drug resistance development, inducing resistance to the anti-proliferative effect of IFN-γ and leading to immune resistance and tumor recurrence (145). In-depth studies have unearthed additional mechanisms, such as deficiencies in antigen presentation (146), depletion of neoantigens (147), and tumor-mediated immunosuppression (148), all contributing to acquired resistance to ICIs and subsequent tumor progression.

Tumor heterogeneity facilitates the emergence of subclones that express different antigens profiles, making them less recognizable to T cells, which contribute to treatment failure. This reinforces the need for ongoing monitoring of tumor antigen profiles during treatment (149). The dynamic nature of the TME contributes to acquired resistance through alterations in immune cell composition over time (150). Changes in myeloid and lymphoid cells within the TME can lead to increased immunosuppressive activity, diminishing the effectiveness of previously effective ICI therapies. For example, while activated effector T cells may initially infiltrate the tumor (151), their numbers may dwindle over time due to the recruitment of immunosuppressive cells, such as MDSCs and Tregs, further diminishing the anti-tumor immune response (152).

Chronic stimulation of T cells in the TME can lead to T cell exhaustion, characterized by the upregulation of inhibitory receptors such as PD-1, CTLA-4, and LAG-3, as well as reduced cytokine production (153). This state of dysfunction complicates future therapeutic interventions aimed at reinvigorating T cell responses. Tumors can activate alternative signaling pathways in response to immunotherapy, affording them survival advantages despite immune pressure. For instance, the aberrant activation of the JAK/STAT pathway can enhance the expression of anti-apoptotic proteins and promote tumor cell survival (16). Changes in signaling within the TME, influenced by both tumor and immune cell interactions, can foster an environment that promotes tumor growth while inhibiting effective immune surveillance (154). Mechanisms such as activation of the NF-κB pathway can further promote survival and proliferation of tumor cells, leading to persistent and ongoing growth despite the presence of ICIs (155).

Tumors can activate alternate signaling pathways (e.g., the JAK/STAT pathway) to promote survival despite immune pressure (32). To maximize the efficacy of ICIs, it is vital to consider non-TME factors such as host genetics and comorbidities in understanding resistance mechanisms. These elements can influence treatment outcomes significantly and require further exploration. Furthermore, comprehensive strategies for combination therapies should delve into the optimal sequencing and timing of ICIs, including the comparison of neoadjuvant versus adjuvant administrations. By integrating knowledge of both primary and acquired resistance mechanisms and defining patient stratification based on genetic and tumor characteristics, future therapeutic strategies can be developed to overcome resistance barriers and improve patient outcomes in ICI therapy.

As ICIs have transformed cancer treatment, the management of irAEs has become an essential aspect of care (156). These adverse effects can range from mild to severe and may involve various organ systems, including the skin, gastrointestinal tract, liver, and endocrine glands. Effective toxicity management strategies include proactive monitoring and early intervention. For instance, corticosteroids are commonly employed to manage severe irAEs such as pneumonitis and colitis. Immune modulation techniques, including the use of ICIs in conjunction with other therapies, may enhance the overall safety profile of treatment regimens (157).

Combination therapies often pose additional risks for irAEs; therefore, prioritizing patient education about potential side effects and providing clear guidelines for reporting symptoms are critical (158). This proactive approach ensures timely interventions and minimizes the impact of irAEs on patients’ quality of life. In conclusion, a comprehensive understanding of resistance mechanisms, including the molecular pathways responsible for primary and acquired resistance and the management of irAEs, is essential for optimizing the therapeutic use of ICIs. By addressing these challenges, we can develop future therapeutic strategies aimed at overcoming resistance and improving patient outcomes in NSCLC and beyond.

## 4 Novel techniques to overcome resistance to immune checkpoint inhibitor therapy

**Figure 5:**
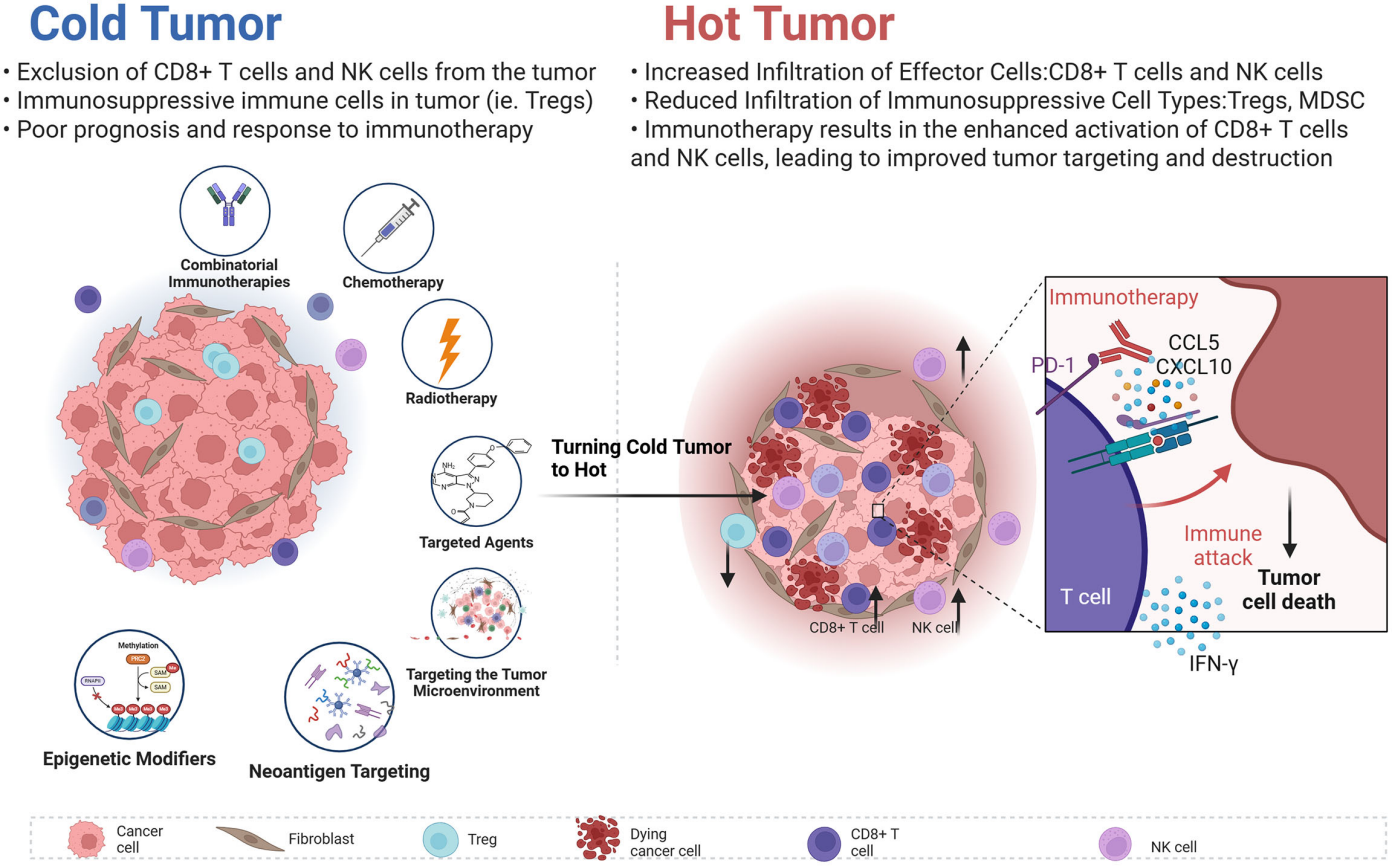
Novel techniques to overcome resistance to immune checkpoint inhibitor therapy.

Recent advances in technology and research have ushered in innovative strategies aimed at overcoming resistance to ICIs (159). This section will discuss not only novel techniques designed to enhance anti-tumor responses through combination therapies and targeting TME but also highlight cutting-edge approaches such as the role of AI in predicting immune responses and the application of microbiome-modulating therapies (**Figure 5**).

For example, Robert D. Schreiber team published findings in Nature indicating that MHC-II neoantigens are crucial in shaping tumor immunity and response to immunotherapy. Effective anti-tumor responses require both CD8^+^T cells recognizing tumor antigens and activated CD4^+^T cells within the TME. These insights affirm MHC class II tumor antigens’ significant role in cancer immunity (160). This section will discuss novel techniques designed to enhance anti-tumor responses through combination therapies, TME targeting, personalized treatment approaches, epigenetic modifiers, and combined immunotherapies.

### 4.1 Innovative strategies in predicting immune responses

AI and machine learning are increasingly being utilized to predict patient responses to ICIs. By analyzing large datasets, these technologies can identify patterns and biomarkers, such as TMB and MSI, which correlate with patient responsiveness to therapy. For example, AI algorithms can assess genomic and transcriptomic profiles of tumors to determine the likelihood of therapeutic success with ICIs, facilitating tailored treatment options for patients (161). Integrating these AI-driven insights into clinical practice may significantly enhance the selectivity and efficacy of ICI therapies (50).

### 4.2 Microbiome regulatory therapies

Emerging evidence also highlights the role of gut microbiota in modulating the effectiveness of ICI treatment and influencing irAEs (162). Microbiome-modulating therapies are being explored as a potential avenue to enhance ICI efficacy (163). The gut microbiota produces various metabolites that can influence immune responses, with short-chain fatty acids (SCFAs), such as butyrate showing promise for their anti-inflammatory properties (164). Research indicates that butyrate-based treatments may enhance ICI outcomes while mitigating side effects. Studies have also identified specific pathways, like the PD-L2-RGMB axis, by which the gut microbiota could promote responses to PD-1 blockade, suggesting an innovative strategy to improve outcomes for patients who do not respond to conventional immunotherapy (165).

### 4.3 Combination therapies

Combining ICIs with other therapeutic modalities has garnered significant attention, effectively enhancing anti-tumor immune responses.

#### 4.3.1 Chemotherapy

Recent findings suggest that specific chemotherapeutic agents can enhance tumor immunogenicity, increasing susceptibility to immune-mediated destruction (166). Chemotherapy-induced immunogenic cell death can facilitate tumor-associated antigen release, priming the immune system for recognition and targeting of cancer cells (167, 168). Analyses of clinical datasets suggest that high TMB and PD-L1 expression can predict favorable T cell responses, whereas specific somatic mutations (e.g., EGFR, KRAS/STK11) correlate with poor T cell response. Ayers et al. developed an immune score that factors in genes associated with the immune response, including IFN-γ signaling and T-cell cytotoxic activity. Investigating immune-related gene landscapes implicated in STING pathway activation has revealed correlations between STING activation and immune checkpoints in NSCLC (169).

For instance, Ultrasound-responsive low-dose doxorubicin liposomes trigger mitochondrial DNA release and activate cGAS-STING-mediated The research paper “antitumor immunity” reveals the significance of oxidized tumor mitochondrial DNA in Sting-mediated anti-tumor immunity and may stimulate the development of more effective cancer immunotherapy strategies (170). A research paper on “photo-triggered self-accelerated nanoplatform for multifunctional image-guided combination cancer immunotherapy” In this study, researchers reported a strategy of developing therapeutic diagnostic agents through reasonable molecular design to enhance anti-tumor immunotherapy. The experimental results show that the combination of PDT and PTX chemotherapy inducing the death of immunogenic cancer cells can not only trigger a strong anti-tumor immunity to inhibit the primary tumor, but also suppress the growth of distant tumors in 4T1 tumor-bearing female mice (171).

Additionally, chemotherapeutic agents like doxorubicin and cyclophosphamide have been shown to increase the expression of tumor neoantigens, enhance MHC expression, and promote T cell infiltration into the TME (172, 173). Qiao Xiaosu et al. reported that doxorubicin activates the STAT1-IRF1-CXCL10 axis, which enhances the efficacy of ICIs (174). Chemotherapy in combination with ICIs amplifies tumor immunogenicity, improving immune checkpoint blockade effectiveness (175, 176). Ongoing clinical trials across various cancer types, including lung cancer (177), melanoma (178, 179), and bladder cancer (180), are evaluating these combinations. Early results demonstrate enhanced overall survival rates and response rates compared to single-agent ICI therapy, such as combining pembrolizumab with chemotherapy in NSCLC (181). Neoadjuvant pembrolizumab plus chemotherapy followed by adjuvant pembrolizumab has shown significant overall survival benefits compared to neoadjuvant chemotherapy alone, indicating the potential of chemotherapy as a powerful ICI adjuvant (**Table 1**).

**Table 1:** Major clinical trials of immune checkpoint inhibitors in NSCLC.

Trial (Phase)	Treatment Arms	Patient Population	Primary Outcomes	Key Findings	References
KEYNOTE-001 (IB)	Pembrolizumab	treatment-naive patients with advanced NSCLC whose tumors expressed PD-L1 (≥1% staining)	**TPS ≥50%**	provides promising long-term OS benefit with a manageable safety profile for PD-L1-expressing treatment-naive advanced NSCLC, with greatest efficacy observed in patients with TPS ≥50%.	(182)
KEYNOTE-024 (III)	Pembrolizumab vs. Platinum Chemo	PD-L1 tumor proportion score (TPS)≥50%, and no targetable EGFR/ALK alterations	**OS:** 43.7 vs. 24.9 mo (HR 0.50, *p*<0.001)	First-line pembrolizumab monotherapy continued to provide durable long-term OS benefit vs chemotherapy despite most patients assigned to chemotherapy crossing over to pembrolizumab. Pembrolizumab was associated with less toxicity than chemotherapy.	(183)
KEYNOTE-189 (III)	Pembrolizumab + Pemetrexed/Platinum vs. Placebo + Chemo	patients with previously untreated metastatic nonsquamous NSCLC without sensitizing *EGFR*/*ALK* alterations, regardless of PD-L1 expression.	**OS:** 31.3 vs. 17.4 mo (HR 0.50; p<0.001)	Chemo-immunotherapy combo benefit regardless of PD-L1 status. Pembrolizumab plus pemetrexed-platinum remains a standard-of-care therapy for patients with newly diagnosed metastatic nonsquamous NSCLC	(184)
CheckMate 227 (III)	Nivolumab + Ipilimumab vs. Chemo	TMB ≥10 mut/Mb (1L mNSCLC)	Symptom deterioration (22.3% versus 35.0%)	First-line nivolumab + ipilimumab demonstrated early, sustained improvements in PROs versus chemotherapy in patients with advanced NSCLC and high TMB.	(185)
IMpower150 (III)	Atezolizumab + Bevacizumab + Chemo (ABCP) vs. BCP	wild-type patients (patients with epidermal growth factor receptor [EGFR] or anaplastic lymphoma kinase [ALK] genetic alterations were excluded	**OS:** 19.2 vs. 14.7 mo (HR 0.78; p=0.02)	Quadruplet therapy (ABCP) improved OS, including in EGFR/ALK+ patients post-TKI. Angiogenesis inhibition synergizes with ICI.	(186)
PACIFIC (III)	Durvalumab vs. Placebo after CRT	Unresectable Stage III NSCLC	**5-yr OS:** 47.5% vs. 29.1% (HR 0.72)	robust and sustained OS and durable PFS benefit with durvalumab after chemoradiotherapy.	(187)
CheckMate 9LA (III)	Nivolumab + Ipilimumab + 2x Chemo vs. Chemo	Patients With Brain Metastases or Select Somatic Mutations	**OS:** 15.8 vs. 11.0 mo (HR 0.74; p<0.001)	further supporting the regimen as first-line treatment for patients with metastatic NSCLC.	(188)
KEYNOTE-598 (III)	Pembrolizumab + Ipilimumab vs. Pembrolizumab + Placebo	PD-L1 TPS ≥50%	**OS:** 22.1 vs. 22.7 mo (HR 1.05; p=0.74)	Pembrolizumab monotherapy remains a standard of care therapy for metastatic NSCLC with PD-L1 TPS ≥50%.	(189)
NEOSTAR (II)	Nivolumab ± Ipilimumab (Neoadjuvant)	Resectable Stage I-IIIA NSCLC	**MPR:** 32.1(Nivo+Ipi) vs. 22% (Nivo)	The dual immune checkpoint therapy plus CT produces numerically higher MPR rates, is overall safe and tolerated, enhances anti-tumor immune activity and mitigates an immunosuppressive phenotype	(190)
SKYSCRAPER-01 (III)	Tiragolumab (anti-TIGIT) + Atezo vs. Atezo	PD-L1 TPS ≥50%	**OS:** 17.9 vs. 16.9 mo, HR=0.87.	Failed to improve OS despite promising early data. Highlights challenges in targeting novel checkpoints.	(191)
LAURA (III)	Osimertinib + Durvalumab vs. Osimertinib (Ongoing)	EGFR-mutant mNSCLC (1L)	*Primary endpoint: PFS*	Aims to overcome ICI resistance in oncogene-driven NSCLC. Early data show manageable safety.	(192)

Bold values indicate statistically significant results (e.g., overall survival [OS], hazard ratio [HR], or other primary endpoints) as reported in the respective clinical trials.

#### 4.3.2 Targeted agents

Integrating targeted therapies with ICIs has shown promise, especially in tumors with well-defined oncogenic mutations. Agents targeting pathways like BRAF and MEK have revolutionized melanoma treatment (193). These therapies can reshape TME, making it more susceptible to immune attacks. For instance, BRAF/MEK inhibitors can mitigate the immunosuppressive milieu often associated with advanced melanoma, facilitating increased T cell infiltration and enhancing anti-tumor responses triggered by ICIs (173, 194). In preclinical models, the combination of BRAF inhibitors with PD-1 blockades has demonstrated synergistic effects, leading to improved survival subsets of patients (195). Early-phase clinical trials have affirmed these results, showed higher response rates and increased overall survival for patients with unresectable metastatic melanoma when treated with combinations.

#### 4.3.3 Radiotherapy

Radiotherapy serves as a promising adjunct to ICI therapy, inducing immunogenic cell death and activating the immune system by catalyzing tumor-associated antigen release (196). By “stirring up” the TME, radiotherapy can enhance the effectiveness of ICIs. Exploring the “abscopal effect”, wherein localized radiotherapy induces systemic anti-tumor effects, provides a strategic avenue for augmenting ICI efficacy (197). Radiotherapy can activate club cells in the lungs, releasing proteins beneficial for immunotherapy, effectively inhibiting MDSCs and reducing pro-cancer inflammation in the TME, ultimately enhancing anti-tumor immune responses and PD-1 inhibitor efficacy (198). Clinical studies evaluating combined radiotherapy with ICIs, such as pembrolizumab or nivolumab, have shown encouraging results, particularly in melanoma and lung cancer, with durable responses and prolonged survival noted (199–204). Ongoing efforts aim to optimize the timing and dosage of radiotherapy alongside ICIs to maximize therapeutic benefit.

### 4.4 Targeting the tumor microenvironment

Understanding the TME is crucial for devising strategies to improve the efficacy of ICIs. The presence of various immunosuppressive cells and factors within the TME can significantly hinder immune responses against tumors. Innovative strategies targeting these immunosuppressive elements may enhance ICI effectiveness.

#### 4.4.1 Depleting immunosuppressive cells

Targeting immunosuppressive cell types, such as Tregs and MDSCs, represents a promising strategy to enhance T cell activity. Therapies that deplete Tregs from the TME can increase effector T cell numbers, promoting a robust anti-tumor response (114, 205). The “active reconnection of SREBP-dependent *de novo* lipid biosynthesis” in Tregs within the TME plays a critical role in maintaining their functional state (206). Similarly, targeting MDSCs has gained attention as a strategy to mitigate immune suppression (207, 208). Agents that inhibit the recruitment or function of MDSCs can enhance effector T cell activation and proliferation, potentially reversing tumor resistance mechanisms (209). Investigational agents, such as anti-CSF-1R and anti-CD47 antibodies, are currently being explored for their ability to target MDSCs and alter the TME in a way that favors immune response (210).

#### 4.4.2 Cytokine-based therapies

Cytokines play a pivotal role in modulating immune responses. Therapeutic cytokines like interleukin-2 (IL-2) and interleukin-12 (IL-12) can drive T cell proliferation and activate anti-tumor immune responses. These cytokines stimulate the proliferation and differentiation of cytotoxic T lymphocytes (CTLs) and NK cells, thereby boosting the immune system’s ability to combat tumors (211). Various clinical trials are currently exploring the concurrent administration of cytokines with ICIs. For instance, combining low-dose IL-2 with PD-1 inhibitors has shown enhanced T cell activation and a favorable safety profile, suggesting a potential synergistic effect that warrants further investigation (212).

A study published in Nature titled “IL-27 Elicits a Cytotoxic CD8^+^ T Cell Program to Enforce Tumor Control” revealed that IL-27 (interleukin-27), an important immune cytokine, has significant potential in regulating immune responses (213). IL-27 not only plays a key role in the growth and differentiation of T cells but also enhances the persistence and efficacy of CD8^+^ T cells within the TME, promoting their immune attack on tumors (214). The role of IL-27 is particularly remarkable because it can both enhance the efficacy of anti-tumor T cells and produce a synergistic effect when used in combination with other ICIs, further improving therapeutic outcomes (213).

### 4.5 Precision medicine and biomarker-driven approaches

The era of precision medicine emphasizes tailoring cancer treatment based on individual patient characteristics, particularly through advancements in genomics and proteomics (215). This personalized approach is critical for developing strategies for ICIs therapies. Identifying predictive biomarkers is essential for optimizing patient selection and improving treatment efficacy. Biomarker-driven approaches allow clinicians to ascertain which patients are most likely to benefit from ICIs, thus minimizing unnecessary toxicity in non-responders (216). Among the most promising predictive biomarkers are TMB and MSI. High TMB is indicative of a greater number of mutations, often leading to the production of more neoantigens, which enhances the likelihood of a robust immune response to ICIs (12). MSI, a condition characterized by the accumulation of insertion or deletion mutations in microsatellite regions of DNA, has also shown predictive value in assessing response to PD-1 blockade (217).

Advancements in genomic, transcriptomic, and proteomic technologies have facilitated comprehensive tumor profiling, enabling the identification of various additional biomarkers. Novel markers, such as immune cell infiltration patterns, particularly tumor-infiltrating lymphocytes (TILs), and specific genetic mutations (e.g., KRAS and STK11), are being actively explored for their potential to predict resistance or response to immunotherapies (218). These insights are fundamental in refining treatment approaches and improving patient outcomes.

Additionally, liquid biopsy techniques that analyze circulating tumor DNA (ctDNA) offer a non-invasive method for real-time monitoring of tumor dynamics and treatment responses (219). This approach allows for continuous evaluation of tumor characteristics, enabling timely adjustments to personalized treatment plans based on evolving biomarker profiles.

#### 4.5.1 Neoantigen targeting

Personalized cancer vaccines targeting tumor-specific neoantigens hold significant promise. Clinical trials exploring personalized neoantigen vaccines have demonstrated their potential to elicit strong immune responses, enhancing the specificity and durability of anti-tumor immunity when combined with ICIs. For example, Autogene Cevumeran, an mRNA-based personalized cancer vaccine, effectively activates T-cell immune responses in patients with advanced solid tumors (220). Neoantigens are unique to cancer cells, arising from tumor-specific mutations, and represent potential targets for T cell recognition. Personalized vaccines can be designed to stimulate a potent T cell response against these neoantigens (36).

Clinical trials exploring personalized neoantigen vaccines, such as neoVax, have shown promise, demonstrating significant immune responses in patients with various malignancies (221). Combining personalized vaccines with ICIs may provide a dual approach to enhance both the specificity and durability of the anti-tumor immune response (36, 222–224). The Phase 1 trial introduced Autogene Cevumeran, an mRNA-based personalized cancer vaccine. By extracting genomic data from patients’ tumor samples, this vaccine identifies and designs personalized treatment plans targeting up to 20 neoantigens. Autogene Coumaran utilizes liposome delivery technology to precisely transport mRNA encoding these neoantigens to dendritic cells within the immune system, thereby activating a T-cell immune response (220). This innovative therapy demonstrated excellent safety and the ability to induce multi-antigen T-cell responses in Phase 1 clinical trials for patients with advanced solid tumors, offering new hope for cancer treatment (220). By extracting genomic data from patients’ tumor samples, personalized vaccines can be designed to stimulate potent T cell responses against these neoantigens. Clinical trials like those involving neoVax have illustrated compelling results, showcasing the potential to induce significant immune responses in various malignancies (221). The inclusion of personalized vaccines combined with ICIs may enhance both the specificity and durability of the anti-tumor immune response, offering new hope for effective cancer treatments (225).

#### 4.5.2 Biomarker-driven strategies

Identifying predictive biomarkers for ICI responsiveness is crucial for optimizing patient selection and improving treatment efficacy. Advances in genomics, transcriptomics, and proteomics allow for comprehensive tumor profiling, revealing potential biomarkers that predict responses to ICIs. For example, high TMB and MSI have emerged as significant biomarkers for predicting response to PD-1 blockade (226, 227). Implementing biomarker-driven strategies can streamline clinical decision-making, ensuring that patients most likely to benefit from ICIs receive the therapy, while minimizing unnecessary toxicity in non-responders (228).

### 4.6 Epigenetic modifiers and their role in precision medicine

Epigenetic modifications can profoundly influence gene expression and contribute to immune evasion. Novel approaches that target these epigenetic alterations hold the potential to enhance sensitivity to ICIs.

#### 4.6.1 Histone deacetylase inhibitors

Histone deacetylase inhibitors are agents that can alter the epigenetic landscape of tumors by preventing the deacetylation of histones, thereby promoting a more permissive chromatin state conducive to gene expression (229). HDAC inhibitors (HDACi) have been shown to induce the expression of immune-related genes, with the potential to reverse immune suppression in the TME. Ongoing clinical studies assessing the combination of HDACi with ICIs suggest that such combinations can restore immune responsiveness and enhance the effectiveness of immune checkpoint blockade (230–232).

#### 4.6.2 DNA methyltransferase inhibitors

DNA methyltransferase inhibitors (DNMTi) aim to reverse abnormal methylation patterns in tumor cells, which can silence key antigens, including neoantigens. By reinstating the expression of these antigens, DNMT inhibitors can improve the immune recognition of tumor cells (233). Investigative studies exploring the synergy between DNMT inhibitors and ICIs point to the potential for enhanced anti-tumor immunity, which is critical for overcoming resistance and expanding the efficacy of immunotherapies (234). Recent advancements in technology and research continue to yield innovative strategies aimed at overcoming resistance to ICI therapy (234). By integrating precision medicine approaches and biomarker-driven strategies, the potential for individualized treatment plans that optimize and enhance responses to immunotherapy become increasingly achievable.

### 4.7 Combinatorial immunotherapies

The combination of different immunotherapeutic agents is another promising avenue for enhancing anti-tumor responses.

#### 4.7.1 Engineered T cell therapies

Chimeric antigen receptor (CAR) T cell therapies represent a groundbreaking approach in oncology, allowing for the engineering of T cells to specifically target tumor-associated antigens (235). When combined with ICIs, such therapies may produce synergistic effects that improve overall therapeutic outcomes (236, 237). Initial clinical trials investigating the combination of CAR T cells and ICIs have shown promising results, demonstrating that these strategies can enhance T cell functionality and promote durable anti-tumor immunity (238, 239).

#### 4.7.2 Immune modulators

The use of immune modulatory agents is another exciting avenue to enhance the effects of ICIs. Agents that activate innate immunity, such as toll-like receptor (TLR) agonists, can boost T cell responses when combined with ICIs (240). Preclinical studies and early-phase clinical trials indicate that these immune modulators can effectively increase T cell activation, proliferation, and cytokine production, paving the way for improved clinical outcomes (241).

In conclusion, the integration of precision medicine and biomarker-driven strategies into ICI therapy represents a paradigm shift in cancer treatment. By tailoring treatment protocols based on comprehensive biomarker assessments, including TMB, MSI, and immune cell infiltration, clinicians can better predict patient responses and customize therapeutic approaches. The exploration of novel strategies, including neoantigen targeting, epigenetic modulation, and microbiome interactions, is crucial for refining cancer immunotherapy and achieving optimal outcomes for patients (242).

While TMB and PD-L1 expression have been established as critical biomarkers in predicting patient responses to ICIs, emerging biomarkers such as T cell receptor (TCR) clonality and microbiome signatures warrant further exploration (243). TCR clonality reflects the diversity of T cell populations within a tumor microenvironment (244). High TCR clonality is often associated with a robust immune response, indicating an active recognition of tumor neoantigens (245). Studies have shown that tumors with high TCR clonality may generate a more effective anti-tumor immune response, as they demonstrate enhanced T cell infiltration and activation (246). Furthermore, TCR sequencing techniques provide an opportunity to profile T cell responses at a granular level, allowing clinicians to identify specific T cell populations that correlate with positive treatment outcomes (247). By integrating TCR clonality analysis into clinical practice, we can better stratify patients and tailor immunotherapy approaches to enhance efficacy.

## 5 Future directions

The evolution of cancer treatment, particularly in the realm of immune checkpoint therapy, has marked significant advancements alongside ongoing challenges. Therapies that block inhibitory pathways in immune cells, such as PD-1/PD-L1 and CTLA-4 antagonists, have notably transformed patient prognoses across various malignancies. However, as we delve deeper into this promising frontier, challenges related to therapeutic resistance and treatment optimization necessitate a multifaceted exploration of future directions. This section highlights key areas for ongoing development, including innovations in AI, ongoing clinical trials, regulatory considerations, patient involvement, and the integration of microbiome therapies.

### 5.1 Artificial intelligence and biomarkers

AI and machine learning (ML) are playing an increasingly pivotal role in cancer treatment, especially in predicting patient responses to ICIs (248). These technologies are set to revolutionize clinical decision-making by analyzing multi-omics data, including genomics, transcriptomics, epigenomics, radiomics, and clinical data, to inform predictions regarding ICI responses, acquired resistance, and the likelihood of irAEs.

By harnessing extensive datasets, AI can provide valuable insights into complex biomarkers like TMB and MSI, guiding clinicians in making informed therapeutic decisions tailored to specific characteristics of patient populations (249). Furthermore, AI-driven methodologies allow for dynamic patient monitoring through advanced techniques, such as CT imaging and radiomics, alongside digital pathology and ctDNA assessments. This holistic integration ensures timely identification of changes in tumor biology, facilitating rapid adjustments to therapy (250).

AI’s potential to optimize personalized treatment options holds vast promise, enabling clinicians to select appropriate interventions—whether single-agent therapies or bespoke combination strategies tailored for specific subsets of patients. Nonetheless, challenges such as data standardization, model interpretability, and the need for prospective validation must be addressed to ensure the reliability and applicability of AI-driven insights in clinical contexts. Large-scale projects utilizing real-world data and biobanks are set to propel the field forward by refining predictive models and validating AI applications across diverse populations, ultimately enhancing our understanding of how to tailor immunotherapy based on specific patient characteristics (251).

### 5.2 Ongoing clinical trials

As our understanding of resistance mechanisms to ICIs, numerous clinical trials are currently underway to investigate combinatorial treatment approaches to overcome these challenges. These trials are critical for evaluating the efficacy and safety of new therapeutic strategies, informing best practices, and optimizing patient management (141).

One promising area of investigation includes combining ICIs with other treatment modalities, such as targeted therapies, chemotherapy, and radiotherapy. Recent clinical trials focusing on PD-1 inhibitors combined with novel agents targeting specific tumor mutations have shown early signs of efficacy (252), emphasizing the importance of personalized medicine. Such combinations leverage distinct mechanisms of action, potentially eliciting robust immune responses while counteracting resistance pathways. However, it is essential to critically assess the feasibility and toxicity of these combinations. For instance, while synergistic effects may be anticipated, the increased complexity of treatment regimens may lead to heightened toxicity profiles, necessitating careful monitoring and management of adverse effects.

Moreover, certain strategies have demonstrated negative data, highlighting the complexities involved in translating preclinical promise into therapeutic benefit. Clinical failures of cytokine therapies illustrate the challenges of achieving meaningful outcomes in a clinical setting (3). Recognizing these limitations enriches our understanding of the current treatment frameworks and informs future research directions by emphasizing the need for robust Phase III data to establish efficacy and safety.

Additionally, novel agents such as bispecific T-cell engagers (BiTEs) and CAR-T therapies are being explored to enhance antitumor activity by more effectively redirecting the immune system against cancer cells (253). However, these promising approaches also raise concerns regarding their feasibility and potential toxicities. The predominance of Phase I/II trial data for these modalities often limits our understanding of long-term outcomes and safety in diverse patient populations. Ongoing trials will be pivotal in revealing how these combinations influence various TMEs and the overall immune landscape, which may ultimately reshape treatment paradigms.

Furthermore, patient stratification is becoming increasingly central to clinical trials as researchers seek to identify specific populations that may benefit most from combination therapies. Biomarkers such as TMB, PD-L1 expression, and T-cell receptor (TCR) diversity will be critical for distinguishing responders from non-responders, thereby enhancing clinical outcomes while reducing unnecessary toxicities associated with ineffective therapies (243).

### 5.3 Regulatory and societal considerations

Translating innovative therapies from research to clinical practice requires robust regulatory frameworks. The rapid pace of advancements in immune checkpoint therapy and combination strategies challenge regulatory bodies tasked with ensuring patient safety while fostering an environment conducive to innovation (254). Regulatory frameworks must adapt quickly to allow timely approval of novel agents and combination therapies that demonstrate promising safety and efficacy profiles.

In evaluating therapies comprising multiple modalities, regulators will need to navigate the complexities of risk-benefit analyses, considering each treatment’s distinct safety profiles and potential toxicities. Cost-effectiveness analysis will be paramount, as the integration of novel therapies can place significant financial burdens on healthcare systems (255). Determining the economic implications of these combinations is essential for assessing their feasibility for widespread adoption within standard clinical practices, especially in publicly funded healthcare systems.

Consequently, enhancing public education regarding these innovative therapies is vital for fostering informed discussions and empowering patients. Initiatives that clearly articulate the potential benefits and risks of new treatments will help cultivate engaged patient communities and ultimately lead to better health outcomes. Navigating these regulatory and societal considerations is essential for successfully implementing immune checkpoint therapies (256).

### 5.4 Patient involvement in research

As cancer research increasingly focuses on patient-centric approaches, involving patients in research discussions has grown critical. Patients provide invaluable insights and experiences that can guide investigators in designing studies that are not only scientifically rigorous but also resonate with real-world needs (257). Incorporating patient perspectives early in the research process can inform the development of clinical trial endpoints that matter most to patients, such as quality of life, symptom management, and long-term survivorship (258). These discussions can direct future investigations to focus on outcomes that align with patient priorities, potentially enhancing participant recruitment and retention rates.

Furthermore, involving patients in study design and execution fosters a sense of ownership and empowerment, encouraging a stronger commitment to research initiatives. Patient advocacy organizations can play instrumental roles in bridging the gap between researchers and the patient community, promoting dialogue that drives innovation and ensures research efforts address pressing challenges within the cancer landscape.

Incorporating patient-reported outcomes (PROs) into clinical trials will be essential for capturing the real-world experiences of patients undergoing immune checkpoint therapy (259). Understanding the psychological, emotional, and social implications of treatment enables a comprehensive understanding of therapeutic efficacy (260). Moreover, PROs can illuminate how combination therapies impact patient well-being, providing invaluable data to inform clinical practice (261). As cancer research continues to evolve, embracing patient involvement as a core component of study design will be paramount. This approach enhances research relevance and fosters the development of therapies that authentically meet the needs of the patients they aim to serve.

## 6 Conclusion

The significant advancements in immune checkpoint therapy represent a remarkable evolution in cancer treatment, offering new hope for patients with previously untreatable malignancies. Nonetheless, the challenges posed by resistance mechanisms necessitate continued exploration and innovation to fully realize the promise of immune checkpoint therapy (262). Future directions must prioritize the exploration and optimization of combination therapies, and embrace insights gained from ongoing clinical trials, which elucidate effective strategies for overcoming resistance. Furthermore, reevaluating regulatory frameworks will be essential for facilitating the translation of novel therapies from the bench to the bedside, ensuring patient safety while promoting timely access to new treatments. Addressing the cost-effectiveness of combination therapies will be crucial for broadening access and ensuring that all patients can benefit equitably from advancements in treatment. Concurrently, fostering patient involvement will remain integral to guiding research efforts toward outcomes that genuinely matter to the cancer community.

Looking ahead, the convergence of novel techniques, personalized approaches, and an enhanced understanding of tumor biology holds the potential to improve patient outcomes. By addressing the challenges of resistance, optimizing therapeutic strategies, and encouraging collaboration among stakeholders, we can pave the way for a transformative era in cancer treatment that fully harnesses the power of immune checkpoint therapy. The path forward is fraught with challenges, but the potential for better patient outcomes is considerable, making this an exciting time in cancer research and treatment.
